# Numerical Simulation of Mandible Bone Remodeling under Tooth Loading: A Parametric Study

**DOI:** 10.1038/s41598-019-51429-w

**Published:** 2019-10-17

**Authors:** Kangning Su, Li Yuan, Jie Yang, Jing Du

**Affiliations:** 1 Department of Mechanical Engineering, Pennsylvania State University, University Park, PA USA; 2 Department of Stomatology Center, Shenzhen People’s Hospital, 2nd Clinical Medical College of Jinan University, Shenzhen, China; 3 Department of Oral Maxillofacial Pathology, Medicine and Surgery, Temple University, Philadelphia, PA USA

**Keywords:** Computational biophysics, Biomedical engineering

## Abstract

Bone adapts to the change of mechanical stimulus by bone remodeling activities. A number of numerical algorithms have been developed to model the adaptive bone remodeling under mechanical loads for orthopedic and dental applications. This paper examines the effects of several model parameters on the computed apparent bone density in mandible under normal chewing and biting forces. The density change rate was based on the strain energy density per unit mass. The algorithms used in this study containing an equilibrium zone (lazy zone) and saturated values of density change rate provides certain stability to result in convergence without discontinuous checkerboard patterns. The parametric study shows that when different boundary conditions were applied, the bone density distributions at convergence were very different, except in the vicinity of the applied loads. Compared with the effects of boundary conditions, the models are less sensitive to the choice of initial density values. Several models starting from different initial density values resulted in similar but not exactly the same bone density distribution at convergence. The results also show that higher reference value of mechanical stimulus resulted in lower average bone density at convergence. Moreover, the width of equilibrium zone did not substantially affect the average density at convergence. However, with increasing width, the areas with the highest and the lowest bone density areas were all reduced. The limitations of the models and challenges for future work were discussed for the better agreement between the computed results and the *in vivo* data.

## Introduction

Bone is a living tissue that adapts to the change of mechanical stimulus by bone remodeling activities^1^, which is a net result of continuous cycles of bone resorption and formation. Many experimental studies have been carried out to study the effects of mechanical stimulus in bone remodeling^2–5^. In addition, a number of numerical algorithms have been developed to model the adaptive bone remodeling under mechanical loads^6–22^. In these models, change of bone mass due to bone remodeling is expressed as a variation in the apparent bone density or in Young’s modulus. Under normal loading conditions, the mechanical stimulus remains at an equilibrium value or in an equilibrium range (dead zone or lazy zone^10,23^) and Young’s modulus or the density of bone remains unchanged. When the mechanical loads change, the mechanical stimulus may shift outside the equilibrium range and lead to increase or decrease of bone modulus or density, which again influence the mechanical stimulus. These cycles persist until the mechanical stimulus returns to the equilibrium value or in the equilibrium range.

In these numerical algorithms, the mechanical stimulus can be strain^6^, effective stress^7–9^, strain energy density per unit bone mass^10–13^, or damage accumulation^14^. The damage accumulation approach was shown to be a special case of the effective stress approach^15^ and also shown to be equivalent to the strain energy density approach under certain conditions^16^. The bone apparent density changing rate or the bone elastic modulus changing rate can be either linear or nonlinear to mechanical stimulus. The rates of increase and decrease can be the same or different.

These algorithms were initially developed for orthopedics applications. And, the results were in good agreement with *in vivo* measurements, especially in the case of femoral head^6–14,16^. The algorithms have been extended to dental problems^17–22^. However, there were still some discrepancies between the computed results and the *in vivo* measurements^17,24,25^. These complex algorithms are iterative, nonlinear and include multiple parameters. Hence, there is a need for a thorough parametric study.

In this study, a parametric study is performed on a bone remodeling algorithm that is based on strain energy density per unit bone mass and is applied to mandibles under normal chewing and biting forces. The effects of several remodeling parameters on the computed overall average apparent bone density and the bone density distribution are studied. These parameters include boundary conditions, initial bone density, reference value of mechanical stimulus, and width of the equilibrium zone. The implications of the results are discussed on the convergence, stability and uniqueness of the algorithms and on the comparison with *in vivo* data.

## Methods

### Strain energy density calculation

**Figure 1:**
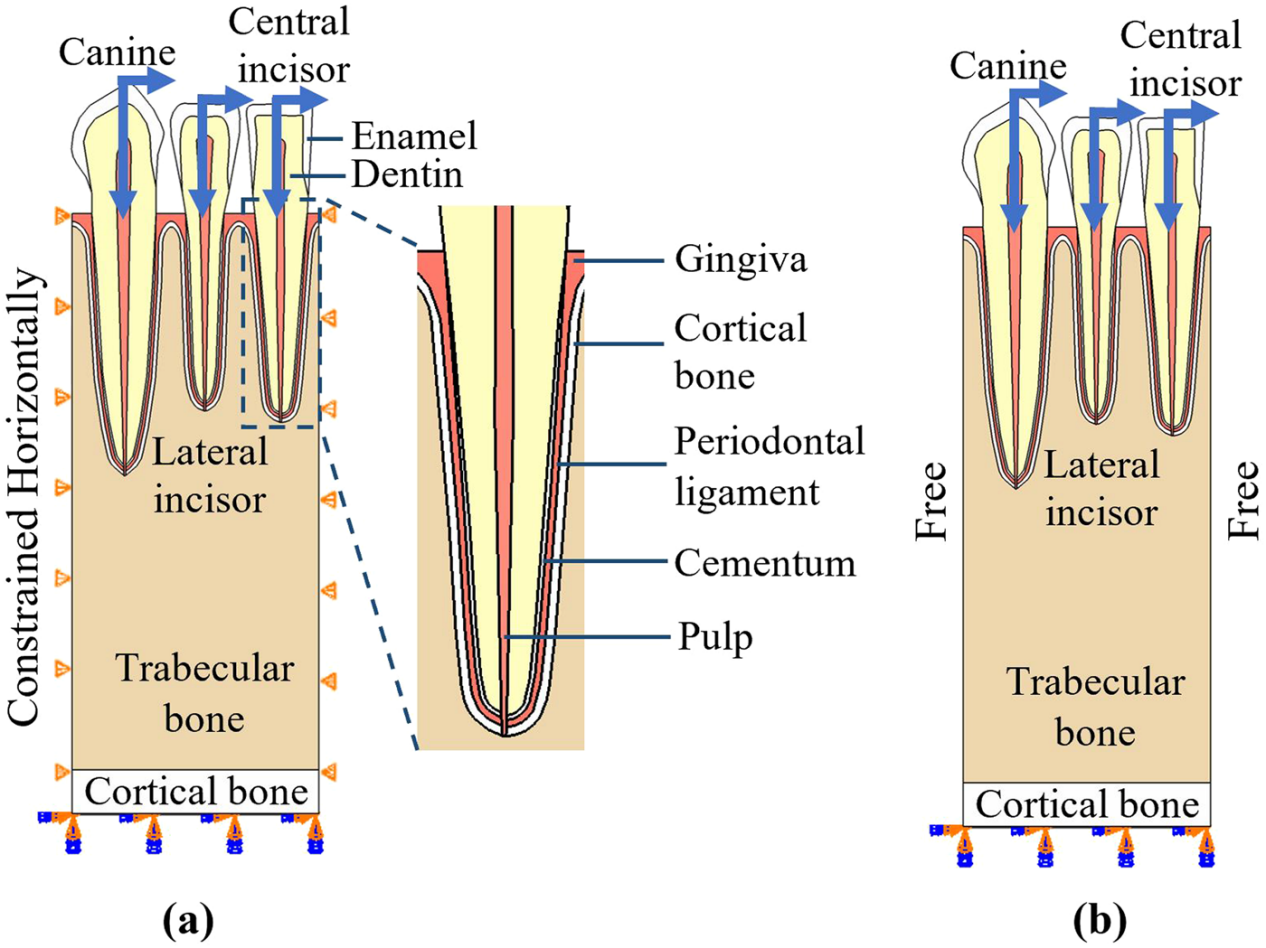
Finite element model of teeth and their surrounding soft and hard tissues for a central incisor, a lateral incisor and a canine. Left and right edges of the models are (**a**) constrained to move only in the vertical direction and (**b**) free to move.

Finite element analysis was carried out to calculate the strain energy density in mandible under normal chewing and biting forces using Abaqus software package (Dassault Systemes Simulia Corporation, Providence, RI). 2-dimensional (2D) models were built to represent the panoramic view of part of a section of mandible, containing three teeth (canine, lateral incisor and central incisor) and their surrounding soft and hard tissues (Fig. 1). The geometry and dimensions of the models were based on the general morphology of natural human teeth^26^. The thickness of cortical bone layer at the bottom of the mandible was set to be 2 mm.

4-node linear quadrilateral elements were used in the mesh. Each model had ~29,000 elements, with ~9,000 of them for the trabecular bone. A load consisting of a vertical component of 100 N and a lateral component of 10 N was applied on each tooth, respectively, to mimic the normal chewing and biting loads (Fig. 1)^27–29^. The bottom of the model was fully fixed. Two types of boundary conditions were used for the left and right edges of the models. They were either constrained to move only in the vertical direction (Fig. 1a) or set to be free to move (Fig. 1b).

These models each consisted of enamel, dentin, pulp, cementum, periodontal ligament (PDL), gingiva, cortical bone and trabecular bone. The material properties used in the simulation for each component in the teeth and their surrounding tissues are listed in Table 1^30^. For simplicity, all the materials were assumed to be linear elastic and isotropic. The interface of PDL-dentin and PLD-bone are bonded as there are no relative movements in between. The relationship between the nominal modulus and apparent density for trabecular bone was measured by Carter and Hayes^31^ and has then adapted^22,32–34^ to be:

1 $$E=C{\rho }^{3}$$

where *E* is the nominal modulus in MPa, *C* is 3790 MPa$\cdot$cm^9^/g^3^ and *ρ* is the apparent density in g/cm^3^. The Young’s modulus for each trabecular bone element was assigned according to the above equation.

**Table 1:** Material properties used in the finite element simulations.

Materials	Enamel	Dentin	Pulp	Cementum	Periodontal Ligament	Gingiva	Cortical Bone
Young’s Modulus (MPa)	79600	18600	150	13700	200	200	13700
Poisson’s ratio	0.3	0.31	0.49	0.3	0.45	0.45	0.3

The strain and stress states in the tooth-bone structures under normal chewing and biting forces were computed using finite element analysis. The total elastic strain energy density, *U*, represents the recoverable part of the energy per unit volume in the element. It is given by:

2 $$U=\frac{1}{2}\sigma \varepsilon $$

where σ and ε are the stress and the strain values for each element, respectively.

### Bone remodeling algorithm

**Figure 2:**
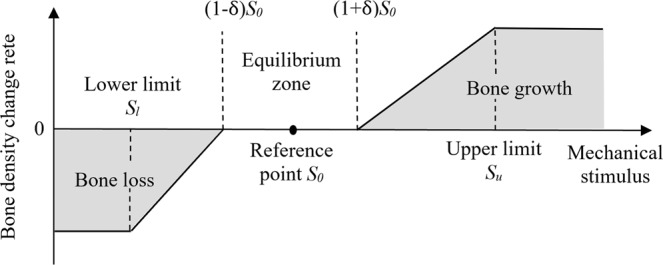
Schematic of bone remodeling rules under mechanical stimulus.

Strain energy density per unit bone mass was chosen to be the mechanical stimulus, S, in the bone remodeling algorithm (Fig. 2). It is given by:

3 $${\rm{S}}=\frac{U}{\rho }$$

The change of apparent bone density in each iteration was calculated using the bone remodeling rules, given by:

4 $$\Delta \rho =\{\begin{array}{lll}-0.05\rho , & bone\,loss\,at\,constant\,rate, & for\,S < {S}_{l}\\ (S-(1-\delta ){S}_{0})B\Delta t, & bone\,loss, & for\,{S}_{l} < \,S < (1-\delta ){S}_{0}\\ 0,\, & equilibrium, & for\,(1-\delta ){S}_{0} < S < (1+\delta ){S}_{0}\\ (S-(1+\delta ){S}_{0})B\Delta t, & bone\,growth, & for\,(1+\delta ){S}_{0} < S < {S}_{u}\\ 0.05\rho , & bone\,growth\,at\,constant\,rate,\, & for\,S > {S}_{u}\end{array}$$

where *S*_0_ is the reference value for the mechanical stimulus, in this case, is the strain energy density per unit bone mass; *S*_*l*_ and *S*_*u*_ are the lower and upper limits of the mechanical stimulus; δ is the half-width of the equilibrium zone; B is the remodeling rate constant; and Δ*t* is the time step. In this study, *B*Δ*t* was chosen to be 2 g^2^/(J · cm^3^). The trabecular bone density was mandated to be higher than 0.1 g/cm^3^ to prevent negative density and lower than 2.2 g/cm^3^, the apparent density of cortical bone^35^.

The bone remodeling algorithm was implemented in customized Python scripts that iterated the above-mentioned finite element analysis. At time 0, the apparent density of trabecular bone was assumed to be uniform. The Young’s modulus for each trabecular bone element was calculated using Eq. (1). The mechanical stimulus in each trabecular bone element under normal chewing and biting forces was calculated using finite element analysis in section 2.1. The apparent density of trabecular bone was updated according to Eq. (4) and then the Young’s modulus was updated using Eq. (1). The process repeated until the averaged bone density in all trabecular bone elements changes by less than 0.03% in the last two iteration steps.

### Parametric study

The effects of model parameters on the resulting trabecular bone density were studied. These parameters include boundary conditions, adjacent teeth, initial bone density, reference value for the mechanical stimulus, and width of the equilibrium zone. Two types of boundary conditions were studied with the left and right edges of the models either constrained in the horizontal direction or set to be free (Fig. 1). The effects of initial bone density were studied for a range of uniform initial bone density values from 0.2 to 2.0 g/cm^3^, with an increment of 0.2 g/cm^3^. Initial bone density of 0.8 g/cm^3^ plus random perturbations within 0.5%, 1%, 2% and 5% were also studied. The effects of reference value for mechanical stimulus were studied for a range of reference value from 0.002 to 0.014 J/g with an increment of 0.002 J/g. The effects of width of equilibrium zone mechanical stimulus were also studied for a range of half-widths from 5% to 45% with an increment of 10%.

When studying the effects of one model parameter, the other parameters remained the same. If not stated specifically, the default setting of model parameters was listed as following: The default models had the left and right edges constrained to move only vertically; The initial bone density was 0.8 g/cm^3^; The reference value of mechanical stimulus was 0.008 J/g; And, the half-width of the equilibrium zone was 15%.

## Results

### Effects of boundary conditions

**Figure 3:**
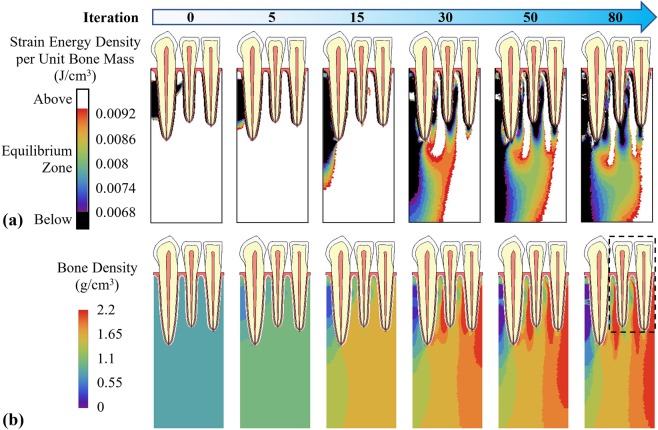
Adaptation of (**a**) mechanical stimulus (strain energy density per unit bone mass) and (**b**) bone density under mechanical stimulus with constrained boundary conditions.

The computed adaptation of apparent bone density and the change of mechanical stimulus (strain energy density per unit bone mass) for a model with constrained boundary conditions are illustrated in Fig. 3. Initially, the mechanical stimulus in most of the trabecular bone elements was higher than the equilibrium zone (Fig. 3a). During the iterations, the overall mechanical stimulus in the whole structure reduced and the area of bone inside the equilibrium zone increased. The distribution of mechanical stimulus changed rapidly in the first few iterations and then the changing rate reduced. The differences in the distributions of mechanical stimulus were not substantial between the 50^th^ and the 80^th^ iteration steps. The apparent density for trabecular bone was set to be uniform at time 0 (Fig. 3b). As time increased, bone density gradually increased in the structure, especially between tooth roots, where strain energy density per unit mass was higher. It also increased near the right (mesial) edge of the model, the direction of the applied lateral forces, whereas bone density near the left (distal) edge reduced.

**Figure 4:**
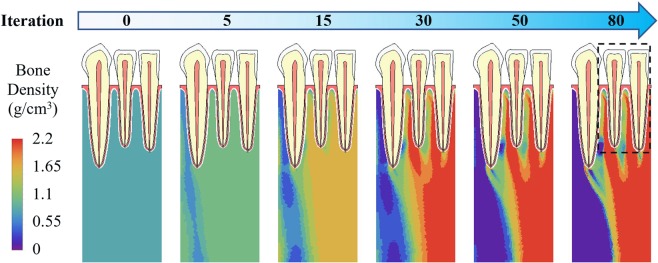
Adaptation of bone density with free boundary conditions.

Instead, when the free boundary conditions were used for the two edges, the computed bone density and mechanical stimulus distributions are illustrated in Fig. 4. Starting with a same uniform density at time 0, bone density also increased between tooth roots and near the right (mesial) edge and decreased near the left (distal) edge over time. However, in the 80^th^ iteration, the bone density distribution was substantially different between the constrained (Fig. 3b) and free (Fig. 4) boundary conditions, especially in the bottom half of the model. At the bottom center of the model, bone density was more uniform with constrained boundaries, but changed drastically for free boundary condition. Only in the vicinity of the teeth (dashed line in Figs 3b and 4), there are some similarities in the bone density distribution.

### Effects of initial bone density

**Figure 5:**
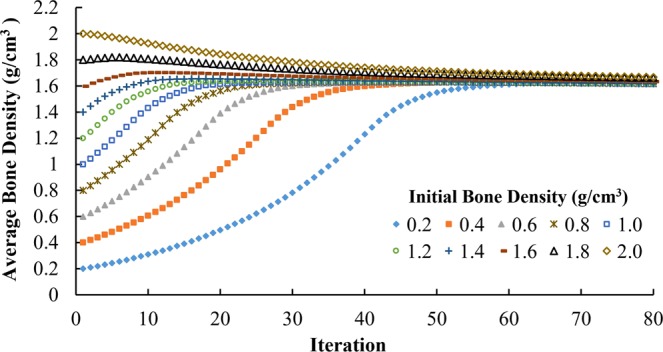
Adaptation of average bone density with uniform initial density ranging from 0.2 to 2.0 g/cm^3^.

**Figure 6:**
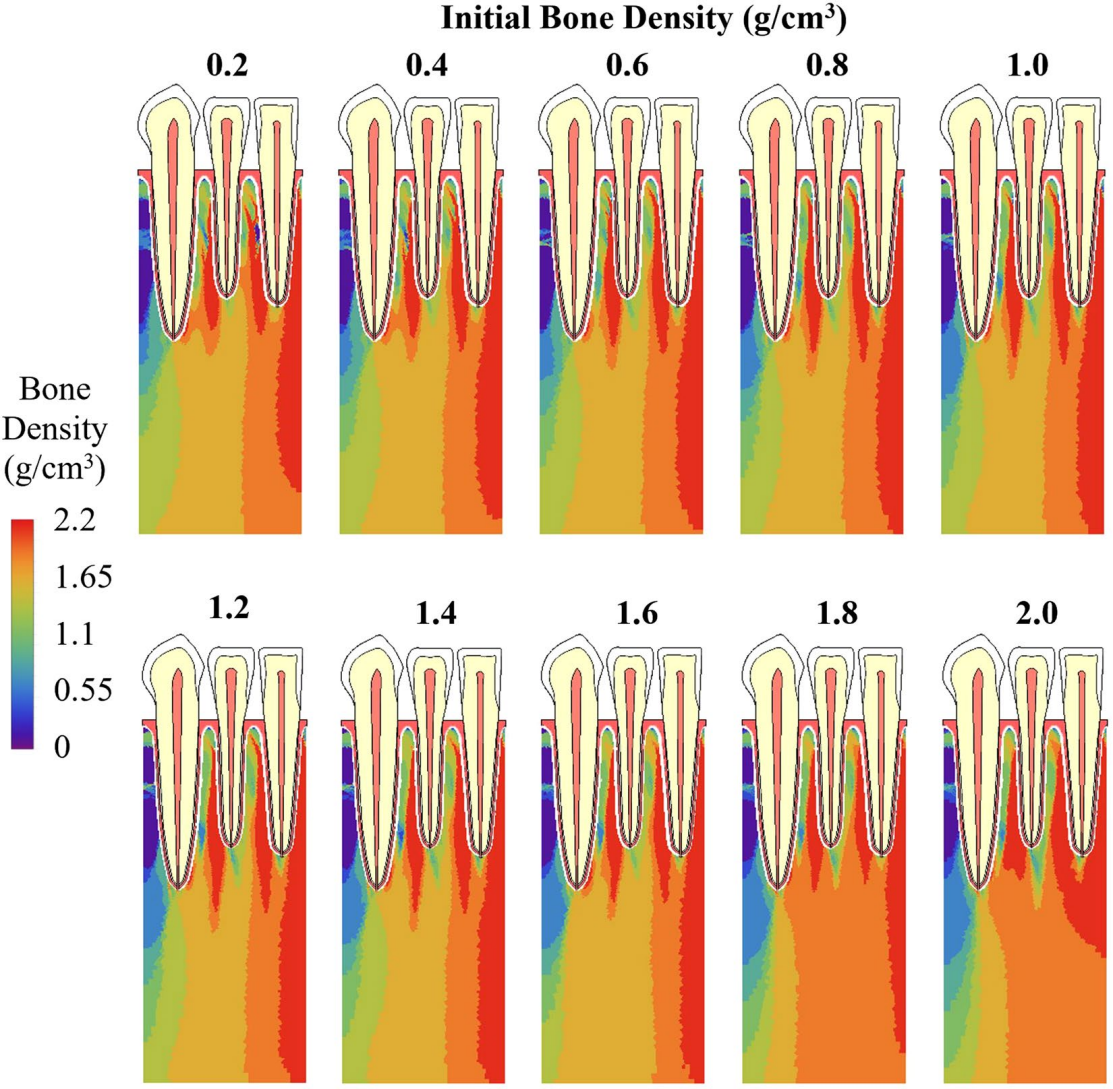
Bone density distribution at the 80^th^ iteration with uniform initial density values ranging from 0.2 to 2.0 g/cm^3^.

**Figure 7:**
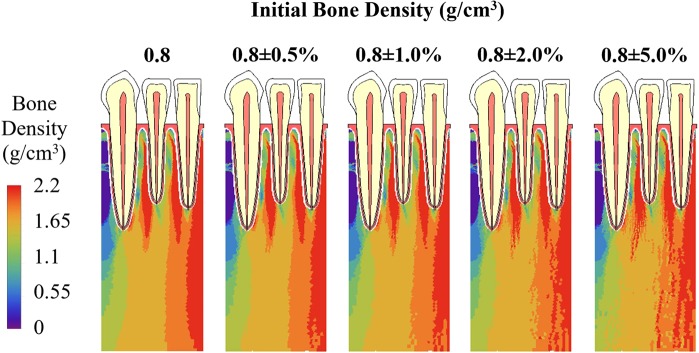
Bone density distribution at the 80^th^ iteration with initial density being 0.8 g/cm^3^ plus a random perturbation within 0.5%, 1%, 2% and 5%.

When the initial bone density at time 0 was equal to or greater than 1.8 g/cm^3^, the average bone density of all trabecular bone elements generally decreased during iterations (Fig. 5). When initial bone density was equal to or lower than 1.6 g/cm^3^, the average bone density increased rapidly in the first few iterations and then changed slowly after it reached a range of 1.6 to1.7 g/cm^3^ (Fig. 5). As time increased, the differences in average bone density resulted from different initial bone densities were getting smaller. The average bone density ranged from 0.2 to 2.0 g/cm^3^ at time 0, 1.55–1.71 g/cm^3^ at the 50^th^ iteration, and 1.62–1.67 g/cm^3^ at the 80^th^ iteration. At the 80^th^ iteration, the differences in the average bone density are negligible (Fig. 5) and the distribution of bone density resulted from different initial bone densities are also very similar (Fig. 6), especially for initial bone densities ranging from 0.2 to 1.8 g/cm^3^. When a small random perturbation was added on the initial density of 0.8 g/cm^3^, the distribution of bone density at the 80^th^ iteration was very similar but not exactly the same (Fig. 7).

### Effects of the reference value of mechanical stimulus

**Figure 8:**
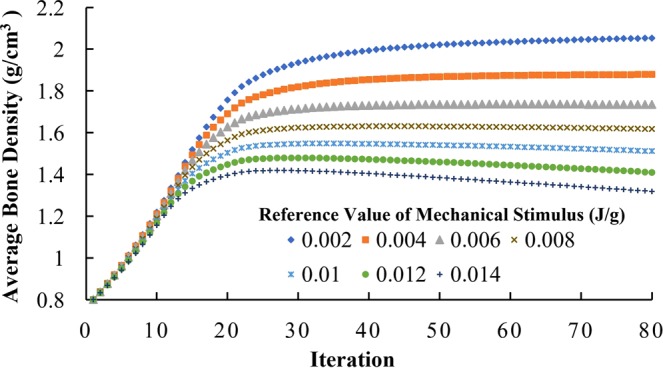
Adaptation of average bone density with different reference values of mechanical stimulus ranging from 0.002 to 0.014 J/g.

**Figure 9:**
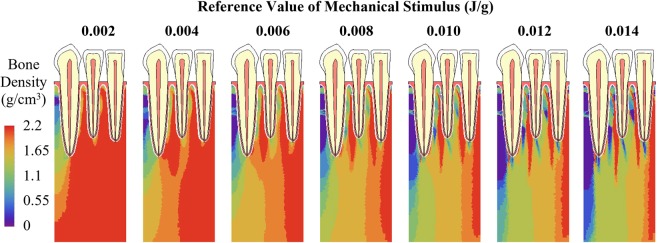
Bone density distribution at the 80^th^ iteration with different reference values of mechanical stimulus ranging from 0.002 to 0.014 J/g.

For different reference values of mechanical stimulus, the average bone densities all increased rapidly in the first 15 iterations at a similar rate (Fig. 8). Then the density change rate greatly reduced, and the average bone densities diverged. At the 80^th^ iteration, for reference values from 0.002 to 0.014 J/g, the average bone density ranged from 2.05 to 1.32 g/cm^3^. Lower reference value of mechanical stimulus resulted in higher average bone density. Generally, the trends in the bone density distribution at the 80^th^ iteration (Fig. 9) were similar, with the density higher towards right (mesial) direction than the left (distal) direction. With increasing reference value, the area with the highest bone density (red) was reduced, but the area with lowest bone density (blue) was enlarged (Fig. 9), which resulted in lower overall average density and was consistent with the trends in Fig. 8.

### Effects of the width of equilibrium zone

**Figure 10:**
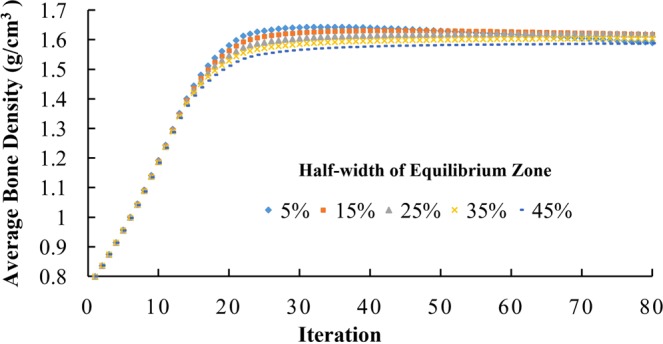
Adaptation of average bone density with different half-widths of equilibrium zone ranging from 5% to 45%.

**Figure 11:**
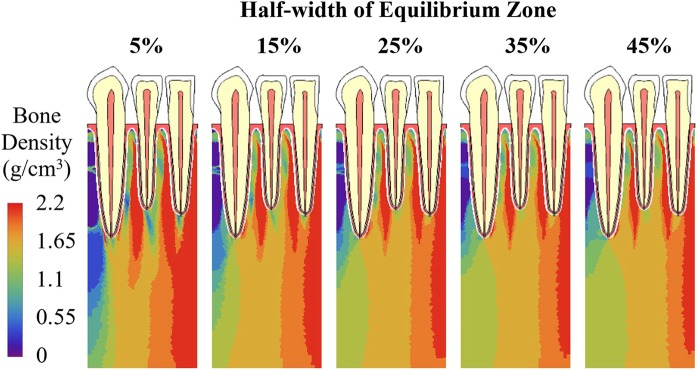
Bone density distribution at the 80^th^ iteration with different half-widths of equilibrium zone ranging from 5% to 45%.

During iterations, in each iteration step, different half-widths of equilibrium zone from 5% to 45% all resulted in similar average bone density (Fig. 10). For different half-widths, the trends in the distribution of bone density at the 80^th^ iteration (Fig. 11) were also similar, with the lowest bone density (blue) and highest bone density (red) at similar locations. However, with increasing half-width, the areas with lowest bone density (blue) and the areas with highest bone density (red) were all reduced. They compensated each other and resulted in similar overall average bone density, as presented in Fig. 10.

## Discussion

### Convergence, stability and uniqueness

In the literatures, the convergence of the numerical models of bone remodeling under mechanical stimulus was usually defined as related to the change of overall bone density in the whole structure^9,17,21,24,36–38^. When the change of overall bone density was not substantial, the model was considered to be converged. Another definition of convergence was given by the number of elements that have fallen in the equilibrium zone^19,39^. Not all bone remodeling algorithms have equilibrium zone. Also, not all elements can enter and stay in the equilibrium zone, due to the instability issue and check-board phenomenon that will be discussed later. Müftü *et al*.^19^ have also shown that before the mechanical stimuli of all elements entered the equilibrium zone at the ~1400^th^ iteration, in the last 92% of iterations, there was no substantial change in the bone density distribution. That may explain why the former definition of convergence is more widely adopted.

To promote convergence, a few approaches can be taken. For the stability of the forward Euler method, the product of remodeling rate and time step, BΔt, has to be small. The bone remodeling rate at cell, tissue and organ levels can be measured from *in vitro* and *in vivo* experiments^40^. The time step in numerical models is usually set as a few days, in which bone density usually won’t change substantially in reality. However, small BΔt value can still not guarantee the convergence. One of the reasons can be attributed to the unrealistic uniform density assumption for the initial condition. It may result in mechanical stimuli that are much lower or higher than the reference value in some regions of the models, which may not exist in reality. Even if they exist, the bone density changing rate may not be linearly proportional to the difference in the mechanical stimuli. Instead, bone density changing rate may saturate when the mechanical stimulus is much different from the reference value. Saturated density changing rate was used in some numerical simulations to limit the density change in single iteration step^38,39,41–44^. In this study, the change of density for each element in single iteration step was mandated to be less than or equal to 5%. The two approaches, small BΔt value and saturated density change rate, can both promote convergence, but they also result in slow convergence.

Another approach to promote convergence is to introduce the equilibrium zone near the reference point of mechanical stimulus and to increase the half-width of equilibrium zone, which loosens the condition of convergence. The results of this study also show that although the half-width of equilibrium zone does not substantially affect the overall average bone density of the structure (Fig. 10), for the same initial density, different half-widths resulted in different spatial distribution of bone density at convergence (Fig. 11).

The convergence of one element is also affected by other elements in the model, especially its adjacent elements. Because not all of the elements enter the equilibrium zone at the same iteration step, and the mechanical stimulus inside one element is affected by the mechanics in other elements, especially its adjacent elements. The results of this study show that some elements entered the equilibrium zone and their bone density remained unchanged, but their mechanical stimuli still changed because of the bone density adaptation in the adjacent elements. Gradually, these elements left the equilibrium zone and their bone density had to change, which may bring them back to the equilibrium zone later again.

An extreme result due to the interplay of multiple elements is the checkerboard phenomenon. Weinans *et al*.^12^ first reported the checkerboard phenomenon due to the instability in certain bone remodeling algorithms. The densities of certain elements increased more rapidly than those in other elements, such that they carried more loads than other elements and resulted in more rapid growth of their bone densities. The self-enhancing feedback resulted in the density of cortical bone in certain elements and zero density in their adjacent elements. Instead of convergence to continuous density distribution, the results diverged to the discontinuous checkerboard pattern of bone density. When choosing different algorithms, generally the more iterations steps it takes, the more severe checkerboard phenomenon it will result in^38,39^.

Weinans *et al*.^12^ also pointed out that the inclusion of an equilibrium zone could alleviate the checkerboard phenomenon. Martínez-Reina *et al*.^45^ suggested that using a saturated density change rate, the density in all elements changed more evenly, which could also mitigate the checkerboard phenomenon^38^. In this study, both equilibrium zone and saturated density change rate are included in the bone remodeling algorithm (Fig. 2) (Eq. 4) and there were only 80 iteration steps. Hence, the results do not exhibit obvious checkerboard phenomenon. Other techniques that can increase the stability and reduce checkerboard phenomenon include integration on nodes instead of elements^9^ and mechanical stimulus based on loading history^39^.

With the absence of the equilibrium zone, theoretically the solution to the bone remodeling algorithm is unique^46^. But the uniqueness is conditional, for example, the instability issues discussed earlier may cause solutions to diverge. Starting from different initial densities, same bone remodeling algorithm could result in different checkerboard density distributions^38^. When the equilibrium zone is introduced in the algorithms, the solutions are not unique, because any mechanical stimulus value in the equilibrium zone can stop the bone adaptation process. Martínez-Reina *et al*. showed that using the algorithms with an equilibrium zone, different initial densities resulted in different density distributions^38^. Their work also showed that saturated density change rate improved the uniqueness of the solution^38^. In this work, the equilibrium zone and saturated density change rate are both applied in the bone remodeling algorithm. For initial density from 0.2 to 2.0 g/cm^3^, the overall average density converged to a similar value around 1.67 g/cm^3^ (Fig. 6). With a relatively narrow equilibrium zone of 15%, the spatial bone density distribution at the 80^th^ iteration was similar for initial density from 0.2 to 1.8 g/cm^3^ (Fig. 8).

### Boundary conditions and applied loads

The results of this study show that boundary conditions strongly affected the bone density distribution and need to be carefully defined. The bone density distribution at the 80^th^ iteration for horizontally constrained (Fig. 3b) and free (Fig. 4) boundary conditions are very different, except in the vicinity of teeth (dashed line), where is near the applied forces and far from the edges where boundary conditions were defined. With free boundary conditions, in the 80^th^ iteration, bone density changes drastically in the bottom center of the model, it is not very likely to occur in reality. Hence, the constrained boundary conditions are used in the rest of the study.

Also, the applied loads should substantially affect bone density distribution. However, the amplitude and direction of the loads applied on teeth during normal chewing and biting activities have been well studied^27–29^. Therefore, the parametric study for applied loads was not conducted.

In the numerical simulations for bone remodeling around teeth or dental implants, different model geometries and boundary conditions have been used. Reina *et al*., Chou *et al*. and Liao *et al*. built 3-dimensional (3D) models of whole mandibles and applied the forces deployed by masticatory muscles through distributed load over the insertion area of each muscle, respectively^17,25,47^. These models have the most accurate geometry and boundary conditions, but their disadvantage is the high computing cost. Hence, in some of these models, coarser mesh was generated in area away from the region of interests to reduce the computing cost^47^. Another approach to reduce computing cost is to build 3D models of a section of mandible and then apply the displacement field calculated from whole mandible model on the boundaries of the sectioned models^45^.

Other simplified model geometries and boundary conditions have also been used. In some 3D models of a section of mandible, the two cut surfaces were fully constrained^18,22,48^. In several 2D sagittal section models of mandibles, the bottom surface was fixed and pressure was applied on the border of the cortical bone to simulate the effects of mandibular flexure^19,21,49^. In a 2D distal–mesial sectional model, the distal and mesial edges were fully constrained^20^. In this study, 2D distal–mesial sectional models were built, and two types of boundary conditions were compared with the distal and mesial edges constrained or free to move. Axisymmetric model around dental implant has also been explored with the vertical displacement of bottom surface eliminated but the perimeter free to move^37^.

In the 3D models of whole mandible with masticatory muscle forces applied^17,25,47^, and in the 3D models of sectioned mandible but with boundary conditions inherited from whole mandible models^45^, the models can compute the bone density distribution in both trabecular and cortical bones using the same remodeling rules. In the simplified models without masticatory muscle forces, usually the models could not compute trabecular and cortical bone density using the same remodeling rules, and a few techniques have been used.

In several works^19,21,37,49^, as well as this study, the region of interest is only in the trabecular bone, and cortical bone was assigned a constant elastic modulus and was not involved in the remodeling simulation. Lin *et al*. used different parameters in bone remodeling algorithms for trabecular and cortical bones, respectively^20^. Li *et al*. assigned a baseline mechanical stimulus to all bone elements to maintain the bone density^18^.

In the numerical simulation of bone remodeling in femurs with and without implants, side plate with different thicknesses at different regions was attached to the front plate in simplified 2D models to account for the structural integrity in 3D and to obtain a good representation of trabecular and cortical bone densities using the remodeling algorithm^12^. A direction for the future work on the numerical simulation of bone remodeling around teeth or implants could be to improve the boundary conditions for the simplified 2D models to account for the masticatory muscle forces and obtain accurate trabecular and cortical bone densities with low computing cost.

### Initial bone density

The results of this study show the adaptation processes of alveolar bone under tooth loading through multiple time steps. However, the results are based on the assumption of uniform initial density, which does not present the reality. Therefore, when interpreting the results, it is more important to focus on the end configuration of bone density distribution at convergence rather than the bone density adaptation process during iteration steps. Starting from unrealistic uniform density assumption, the process before convergence does not have great significance for the study of real bone adaptation, but is meaningful for the study of numerical algorithms.

Compared with the effects of boundary conditions, the average bone density and the distribution of bone density at the 80^th^ iteration are not as sensitive to the choice of initial density. Different initial bone density values resulted in very similar average bone density (Fig. 5) and very similar bone density distribution (Figs 6 and 7), especially in the vicinity of the applied loads, i.e. around the roots of teeth. Weinans *et al*. had similar discoveries in their adaptive bone remodeling simulation models for femurs^12^.

### Implications

**Figure 12:**
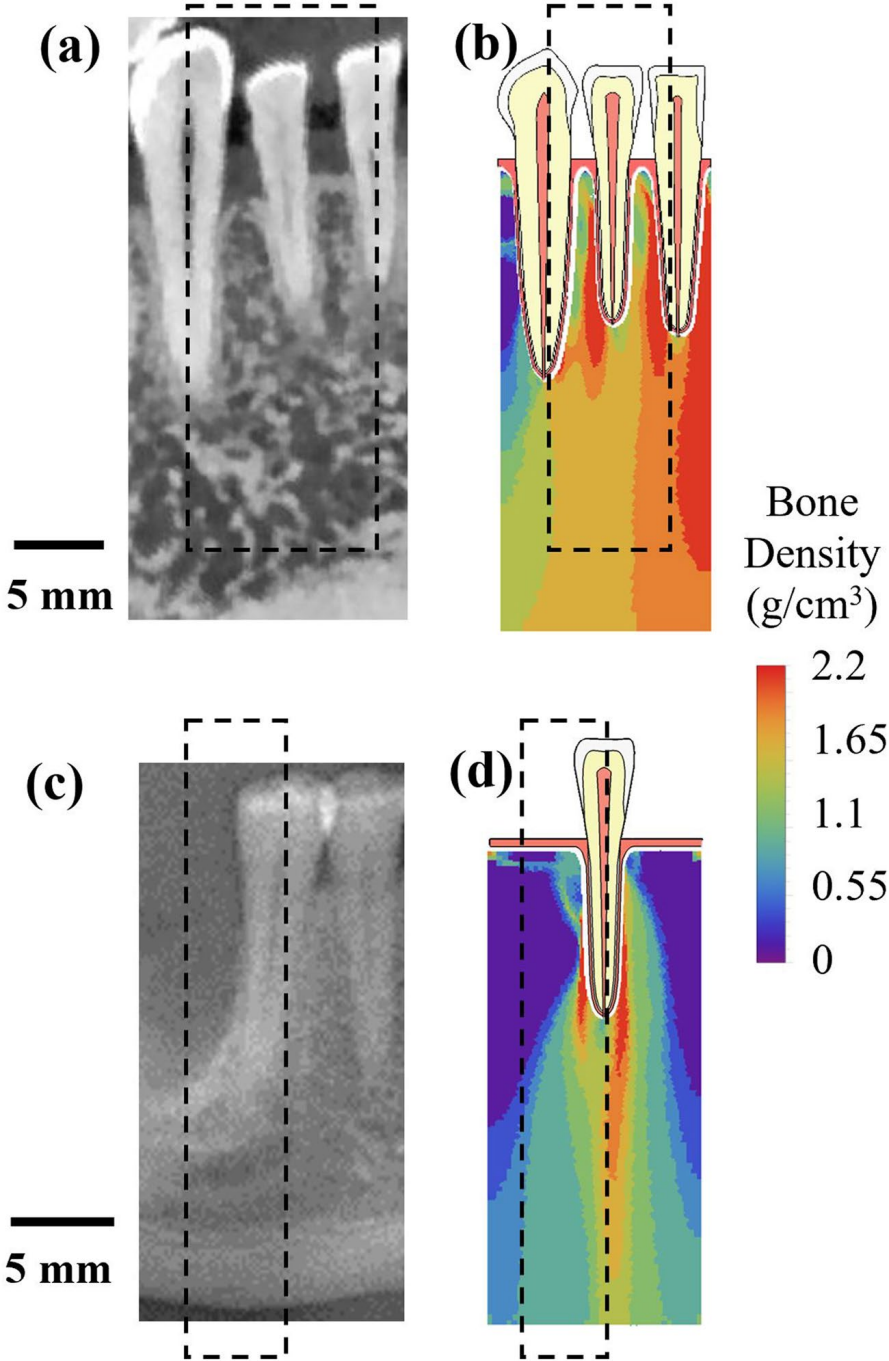
Comparison of cone beam computed tomography (CBCT) images and computed bone density distribution: (**a**) a mesial-distal CBCT virtual section for a region of mandible containing canine, lateral incisor, central incisor; (**b**) computed bone density distribution at the last iteration step for the 3-tooth model; (**c**) a mesial-distal CBCT virtual section for a section of mandible with a missing tooth; (**d**) computed bone density distribution at the last iteration step for a single-tooth model.

The bone density distributions at the last iteration are compared with cone beam computed tomography (CBCT) images in Fig. 12. In the mesial-distal CBCT virtual section in Fig. 12a for a human mandible with canine, lateral incisor and central incisor, trabecular bone between tooth roots is less porous than that below the teeth. The models in this work did not result in porous patterns for trabecular bone but a trend of apparent trabecular bone density. With constrained boundary conditions, at the last iteration, the computed bone density between tooth roots is higher than that below the teeth (Fig. 12b). A mesial-distal CBCT virtual section of a human mandible with a missing tooth is presented in Fig. 12c. Bone adaptation under normal chewing and biting in a single-tooth model was also computed, starting from uniform initial density assumption. The bone density distribution at convergence (Fig. 12d) near the tooth root was higher than that away from the tooth root. The computed bone density at the upper left and upper right parts was close to 0 (Fig. 12d). Clinical studies have shown that after the healing period for tooth extraction, bone level decreases rapidly in the first 6 to 12 months^50^. The current work suggests that bone level decrease can be attributed to the chewing force applied on the remaining teeth, which can only be transmitted to the trabecular bone that is close to the roots.

There are several possible directions for future work. Besides the bone density at convergence, if ones also want to simulate the bone adaptation process before convergence, there is clearly a need to improve the initial condition to better reflect the real bone density distribution in mandible. For example, to simulate the bone adaptation process after implant placement, the initial condition should better represent bone density distribution before implant placement. Moreover, upon improvement of boundary conditions to include the effects of masticatory muscle forces, it is likely that the models will be able to also simulate the adaptation of cortical bone. Also, there is a need to develop quantitative comparison between simulation results and experimental measurements. Furthermore, bone remodeling process contains multiple aspects, including biological, chemical, and mechanical factors. It is affected by gender, aging, disease, injury and treatment. There are also other limitations in the current models. These are clearly some challenges for future work to improve current bone remodeling algorithms and to incorporate these factors.

## Conclusions

This paper presents the results of a parametric study for numerical models of mandible bone adaptive remodeling under normal chewing and biting forces. Bone density increased at the regions where the mechanical stimulus (strain energy density per unit bone mass) was greater than the reference value, and *vice versa*. With the initial density being set uniform, during the iterations, the variations in mechanical stimulus in the whole structure reduced and the variations in the bone density distribution increased.

The parametric study shows that when different boundary conditions were applied, the bone density distribution became very different, except in the vicinity of teeth and applied loads. The results also show that models starting from different initial density values resulted in a similar overall average density and similar bone density distribution at convergence. Higher reference values of the mechanical stimulus resulted in lower overall average bone density. The width of equilibrium zone didn’t substantially affect the average density at convergence. However, with increasing width, the trabecular bone areas with highest or lowest density were all reduced.

The results show that the models used in this study provided certain stability and convergence and did not result in discontinuous checkerboard patterns in the limited number of iteration steps that were carried out in this work. These models still have many limitations, such as the nonrealistic initial conditions, over-simplified boundary conditions and the absence of biological signals. Despite those, the current work provided some guidance for applying the nonlinear multi-parameter bone remodeling algorithms to predict mandible bone density distribution under mechanical loading.
